# Comparison of the new Exponential Injury Severity Score with the Injury Severity Score and the New Injury Severity Score in trauma patients: A cross-sectional study

**DOI:** 10.1371/journal.pone.0187871

**Published:** 2017-11-09

**Authors:** Spencer C. H. Kuo, Pao-Jen Kuo, Yi-Chun Chen, Peng-Chen Chien, Hsiao-Yun Hsieh, Ching-Hua Hsieh

**Affiliations:** Department of Plastic and Reconstructive Surgery, Kaohsiung Chang Gung Memorial Hospital and Chang Gung University College of Medicine, Kaohsiung, Taiwan; National Yang-Ming University, TAIWAN

## Abstract

**Objective:**

To compare Exponential Injury Severity Score (EISS) with Injury Severity Score (ISS) and New Injury Severity Score (NISS) in terms of their predictive capability of the outcomes and medical expenses of hospitalized adult trauma patients.

**Setting:**

This study was based at a level I trauma center in Taiwan.

**Methods:**

Data for 17,855 adult patients hospitalized from January 1, 2009 to December 31, 2015 were retrieved from the Trauma Registry System. The primary outcome was in-hospital mortality. Secondary outcomes were the hospital length of stay (LOS), intensive care unit (ICU) admission rate, ICU LOS, and medical expenses. Chi-square tests were used for categorical variables to determine the significance of the associations between the predictor and outcome variables. Student t-tests were applied to analyze normally distributed data for continuous variables, while Mann-Whitney U tests were used to compare non-normally distributed data.

**Results:**

According to the survival rate-to-severity score relationship curve, we grouped all adult trauma patients based on EISS scores of ≥ 27, 9–26, and < 9. Significantly higher mortality rates were noted in patients with EISS ≥ 27 and those with EISS of 9–26 when compared to patients with EISS < 9; this finding concurred to the findings for groups classified by the ISS and NISS with the cut-off points set between 25 and 16. The hospital LOS, ICU admission rates, and medical expenses for patients with EISS ≥ 27 and patients with EISS of 9–26 were also significantly longer and higher than that of patients with EISS < 9. When comparing the demographics and detailed medical expenses of very severely injured adult trauma patients classified according to ISS, NISS, and EISS, patients with ISS ≥ 25 and NISS ≥ 25 both had significantly lower mortality rates, lower ICU admission rates, and shorter ICU LOS compared to patients with EISS ≥ 27.

**Conclusions:**

EISS 9 and 27 can serve as two cut-off points regarding injury severity, and patients with EISS ≥ 27 have the greatest injury severity. Additionally, these patients have the highest mortality rate, the highest ICU admission rate, and the longest ICU LOS compared to those with ISS ≥ 25 and NISS ≥ 25, suggesting that patients with EISS ≥ 27 have the worst outcome.

## Introduction

Trauma patients present to the emergency department (ED) with a great variety of injuries and diseases. To address these, the Abbreviated Injury Scale (AIS) system defines the severity of injury throughout the different regions of the body. It is an anatomically based, consensus derived, global severity scoring system that classifies an individual injury by body region according to its relative severity on a 6-point scale (1 = minor and 6 = maximal). The system is constantly revised, expanded, and improved, and the Association for the Advancement of Automotive Medicine recently announced its latest revision, the AIS 2005—Update 2008 and AIS 2015. To summarize a single patient’s multiple injures into a single score, the Injury Severity Score (ISS) was created by Baker et al. in 1974, which has been considered the “gold standard” among anatomic injury severity indicators. It is based on the AIS severity values, that is, the summation of the squares of the severity digit in the AIS of the most severe injuries, in three of six predefined body regions[1].

However, the ISS only includes one injury in each body region, which leads to possible inclusion of a less severe injury in other body regions rather than another serious injury in the same body region. To overcome this limitation, a modified ISS, the New Injury Severity Score (NISS) was introduced by Osler et al. in 1997. NISS is simply the sum of squares of the three most severe injuries, regardless of the body regions injured[2]. Further, Wang et al. have created the Exponential Injury Severity Score (EISS) in 2014 by modifying the AIS system. The EISS was computed as the simple change in AIS values by raising each AIS severity score (1–6) by 3 taking a power of AIS minus 2, and then summing the three most severe scores (i.e., highest AIS values), regardless of body regions. If there is an AIS code with a severity of 6 anywhere in the body, other injury body regions of the AIS scores is not calculated. When the AIS score is 2, total number of AIS 2 should be deleted from the total scores. Mathematical expression: EISS = 3^A - 2^ + 3^B - 2^ + 3^C - 2^, where A, B, and C are the three most severe AIS codes (Table 1)[3]. With this exponential transformation of the AIS values, the EISS is expected to be more reflective of the true severity of injuries in a patient with polytrauma. In Wang’s study, the EISS is reported to be more predictive of survival; therefore, it might be used as the standard summary measure of human trauma[3].

**Table 1:** Calculation of Exponential Injury Severity Score (EISS) according to the Abbreviated Injury Scale (AIS) codes.

AIS codes (A)	3^A-2^ (B)	(C)	(D)
1	3^1−2^	3^−1^	0.3
2	3^2−2^	3^0^	1
3	3^3−2^	3^1^	3
4	3^4−2^	3^2^	9
5	3^5−2^	3^3^	27
6	3^6−2^	3^4^	81

A comparison between the ISS and the NISS in terms of their predictive capability for mortality has been conducted and can be found in the literature[4–18]. However, it is not clear whether ISS or NISS is a better predictor of mortality. Moreover, the new EISS has not been applied in real time thus far; therefore, a comparison with other severity scores has not been performed. In this study, we aimed to investigate the three different injury severity scores (the ISS, NISS, and EISS) in terms of their predictive capability of the outcomes and medical expenses of adult trauma patients.

## Methods

We designed a retrospective study reviewing the data for all adult patients aged ≥ 20 years in the Trauma Registry System of Kaohsiung Chang Gung Memorial Hospital from January 2009 to December 2015. This is a 2,686-bed facility and a level I trauma center that provides care to trauma patients primarily from South Taiwan [19, 20]. Detailed patient information was retrieved, including age, gender, AIS, ISS, NISS, hospital length of stay (LOS), intensive care unit (ICU) LOS, in-hospital mortality, and medical expenses. The final score of AIS was be provided for trauma patients after admission and before discharge, but not only according to the situations identified from the first assessment at emergency department. This study was approved by the institutional review board (IRB) of the Kaohsiung Chang Gung Memorial Hospital (IRB approved No. 201600225B0). Informed consent was waived according to IRB regulations. Furthermore, the data were analyzed anonymously so that there was no consent.

In this study, patients were categorized by their ISS, NISS, and EISS. The survival, hospital LOS, and medical expense curves were created. Medical expenses per person included the cost of operation (cost of operation and operation supplies), the cost of examination (cost of physical examination, hematology testing, radiography, pathological examination, electrocardiography, echo, endoscopy, electromyography, cardiac catheterization, and electroencephalography), cost of pharmaceuticals (cost of pharmacy service, medicine, and narcotic drugs), and other medical costs (cost of registration and administration, ward fees, nursing fee, hemodialysis fees, anesthesia fees, rehabilitation-treatment fee, special material costs, and personal expenses). To evaluate the clinical outcome and medical expenses of trauma patients, the severely injured patients (ISS ≥ 25, NISS ≥ 25, and EISS ≥ 27) and the moderately injured patients (ISS of 16–24, NISS of 16–24, and EISS of 9–16) were compared with the mildly injured patients (ISS < 16, NISS < 16, and EISS < 9), using the SPSS v.20 statistical software (IBM, Armonk, NY). Chi-square tests were used for categorical variables to determine the significance of the associations between the predictor and outcome variables. Student t-tests were applied for continuous variables to analyze normally distributed data, while Mann-Whitney U tests were used to compare non-normally distributed data. The corresponding crude odds ratios (ORs) with 95% confidence intervals (CIs) for each variable were obtained. All of the results were presented as the mean ± standard deviation. A *p*-value of < 0.05 was considered statistically significant.

## Results

**Fig 1:**
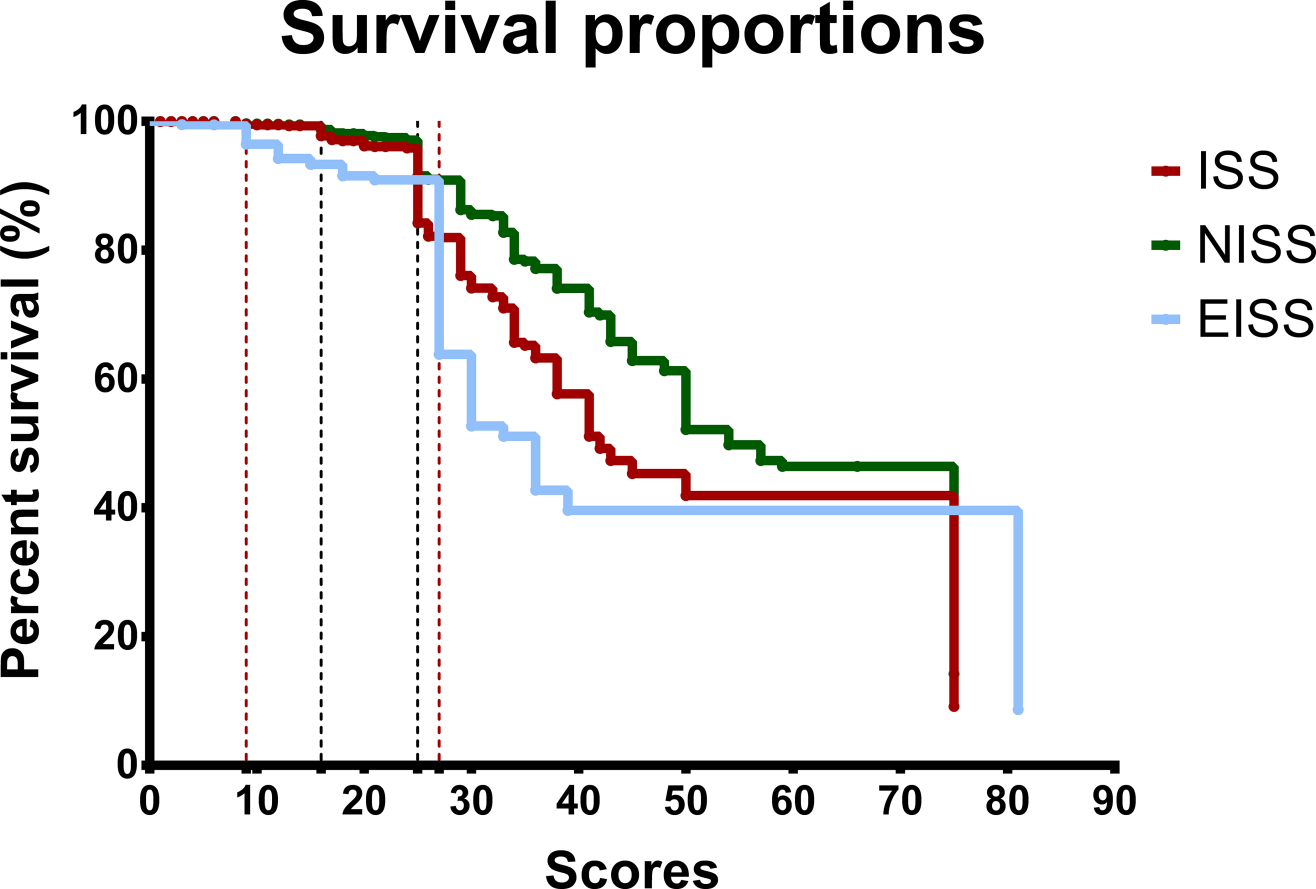
The survival rate-to-severity score relationships of all patients classified according to the ISS, NISS, and EISS.

**Fig 2:**
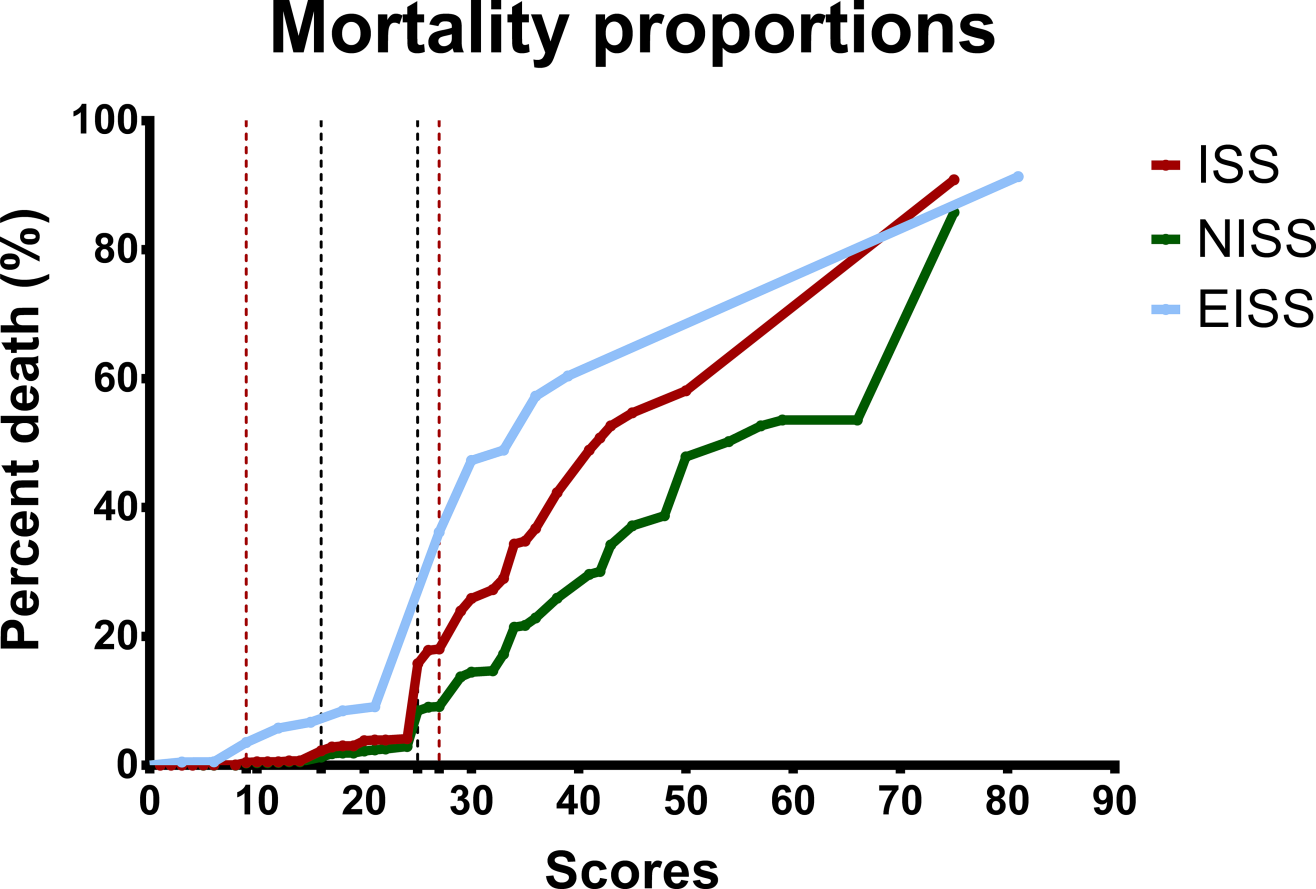
The mortality rate-to-severity score relationships of all patients classified according to the ISS, NISS, and EISS.

According to the survival rate-to-severity score relationships (Fig 1) and mortality rate-to-severity score relationships (Fig 2) of all patients classified according to the ISS, NISS, and EISS, decreases in the survival of patients can be identified at severity scores of 16 and ≥ 25 for patients classified by the ISS and NISS scoring systems. This is compatible with severe and very severe injury according to the literature[21–23] (Fig 1). Meanwhile, decreases in survival are located at severity scores of 9 and ≥27 for patients classified with the EISS. Hence, according to the survival rate-to-severity score relationship curve, we grouped and analyzed all adult trauma patients as patients with EISS ≥ 27, EISS of 9–16, and EISS < 9. Fig 2 shows that mortality increases at the severity scores of 16 and ≥ 25 for patients classified based on ISS and NISS. Mortality increases at the severity scores of 9 and ≥ 22 for patients classified based on EISS.

Relevant demographics and detailed medical expenses of all adult trauma patients classified according to the ISS are summarized in Table 2. Higher mortality rates were noted in patients with ISS ≥ 25 and in patients with ISS of 16–24 when compared to patients with ISS < 16 (OR 122.6 [95% CI 89.13–168.77], *p* < 0.001 and OR 12.1 [95% CI 8.35–17.44], *p* < 0.001, respectively). Similarly, patients with ISS ≥ 25 and ISS of 16–24 had higher ICU admission rates than patients with ISS < 16. Patients with ISS ≥ 25 and ISS of 16–24 also had higher costs of operation, costs of examination, pharmaceutical costs, and total medical expenses compared with patients with ISS < 16 did.

**Table 2:** Demographics and medical expenses of adult trauma patients according to ISS classification.

Variables	ISS≥25 n = 757 (I)	ISS of 16–24 n = 1788 (II)	16>ISS n = 15310 (III)	*OR(95%CI) p*		*OR(95%CI) p*	
				I vs III		II vs III	
Age	51.5±18.8	54.6±19.1	52.2±19.3	—	0.334	—	<0.001
Gender, n (%)					<0.001		<0.001
Male	522(69.0)	1173(65.6)	8408(54.9)	1.8(1.56–2.13)		1.6(1.41–1.74)	
Female	235(31.0)	615(34.4)	6902(45.1)	0.5(0.47–0.64)		0.6(0.58–0.71)	
Mortality, n (%)	217(28.7)	68(3.8)	50(0.3)	122.6(89.13–168.77)	<0.001	12.1(8.35–17.44)	<0.001
Hospital LOS (days)	20.6±19.6	14.3±13.0	8.3±8.2	—	<0.001	—	<0.001
ICU patients, n (%)	664(87.7)	1197(66.9)	1340(8.8)	74.4(59.49–93.14)	<0.001	21.1(18.85–23.65)	<0.001
ICU LOS (days)	12.0±14.8	7.3±9.8	8.5±10.0	—	<0.001	—	0.003
Cost of operation	1148±1530	539±860	434±499	—	<0.001	—	<0.001
Cost of examination	578±612	346±422	104±221	—	<0.001	—	<0.001
Cost of pharmaceutical	919±1608	422±1634	126±437	—	<0.001	—	<0.001
Medical expenses	9652±10694	4754±6165	2538±2945	—	<0.001	—	<0.001

CI = confidence interval; ICU = intensive care unit; ISS = injury severity score; LOS = length of stay; OR = odds ratio

Table 3 summarizes all adult trauma patients classified and analyzed according to the NISS. Patients with higher NISS also had significantly higher mortality rates, longer hospital LOS, and higher ICU admission rates. In terms of medical expenses, patients with NISS ≥ 25 and NISS of 16–24 had significantly higher costs of operation, costs of examination, pharmaceutical costs, and total medical expenses compared to patients with NISS < 16.

**Table 3:** Demographics and medical expenses of adult trauma patients according to NISS classification.

Variables	NISS≥25 n = 1036 (I)	NISS of 16–24 n = 2149 (II)	16>NISS n = 14670 (III)	*OR(95%CI) p*		*OR(95%CI) p*	
				I vs III		II vs III	
Age	52.1±19.0	53.1±19.1	52.3±19.3	—	0.762	—	0.081
Gender, n (%)					<0.001		<0.001
Male	709(68.4)	1365(63.5)	8029(54.7)	1.8(1.57–2.05)		1.4(1.31–1.58)	
Female	327(31.6)	784(36.5)	6641(45.3)	0.6(0.49–0.64)		0.7(0.63–0.76)	
Mortality, n (%)	235(22.7)	56(2.6)	44(0.3)	97.5(70.13–135.61)	<0.001	8.9(5.98–13.24)	<0.001
Hospital LOS (days)	19.5±18.3	14.2±12.5	8.0±7.8	—	<0.001	—	<0.001
ICU patients, n (%)	844(81.5)	1175(54.7)	1182(8.1)	50.2(42.42–59.32)	<0.001	13.8(12.41–15.27)	<0.001
ICU LOS (days)	11.1±13.9	7.0±9.5	8.9±10.4	—	<0.001	—	<0.001
Cost of operation	1022±1420	629±945	414±442	—	<0.001	—	<0.001
Cost of examination	540±580	298±402	99±211	—	<0.001	—	<0.001
Cost of pharmaceutical	803±1472	374±1499	119±422	—	<0.001	—	<0.001
Medical expenses	8683±9875	4816±5986	2408±2686	—	<0.001	—	<0.001

CI = confidence interval; ICU = intensive care unit; ISS = injury severity score; LOS = length of stay; NISS = new injury severity score; OR = odds ratio

The demographics and medical expenses of all adult trauma patients classified according to the EISS are demonstrated in Table 4. As expected, significantly higher mortality rates were noted in patients with EISS ≥ 27 and in patients with EISS of 9–26 when compared to patients with EISS < 9 (OR 235.7 [95% CI 168.78–329.23], *p* <0.001 and OR 17.9 [95% CI 12.73–25.07], *p* <0.001, respectively), which was compatible with the ISS and NISS classifications. The hospital LOS, ICU admission rates, and medical expenses for patients with EISS ≥ 27 and patients with EISS of 9–26 were also significantly longer and higher than that of patients with EISS < 9.

**Table 4:** Demographics and medical expenses of adult trauma patients according to EISS classification.

Variables	EISS≥27 n = 416 (I)	EISS of 9–26 n = 1780 (II)	9>EISS n = 15659 (III)	*OR(95%CI) p*		*OR(95%CI) p*	
				I vs III		II vs III	
Age	52.4±19.3	54.8±19.0	52.1±19.3	—	0.789	—	<0.001
Gender, n(%)					<0.001		<0.001
Male	286(68.8)	1178(66.2)	8639(55.2)	1.8(1.45–2.21)		1.6(1.43–1.76)	
Female	130(31.3)	602(33.8)	7020(44.8)	0.6(0.45–0.69)		0.6(0.57–0.70)	
Mortality, n(%)	183(44.0)	100(5.6)	52(0.3)	235.7(168.78–329.23)	<0.001	17.9(12.73–25.07)	<0.001
Hospital LOS (days)	20.4±22.3	14.9±13.6	8.5±8.4	—	<0.001	—	<0.001
ICU patients, n(%)	384(92.3)	1292(72.6)	1525(9.7)	111.2(77.25–160.13)	<0.001	24.5(21.83–27.58)	<0.001
ICU LOS (days)	14.5±17.8	7.6±9.8	8.4±9.7	—	<0.001	—	0.035
Cost of operation	1332±1789	554±906	443±515	—	<0.001	—	<0.001
Cost of examination	606±665	378±451	110±231	—	<0.001	—	<0.001
Cost of pharmaceutical	1113±1787	464±1688	133±458	—	<0.001	—	<0.001
Medical expenses	10933±12420	5130±6708	2618±3083	—	<0.001	—	<0.001

CI = confidence interval; ICU = intensive care unit; ISS = injury severity score; LOS = length of stay; EISS = exponential injury severity score; OR = odds ratio

The comparison of the demographics and detailed medical expenses of very severely injured adult trauma patients among the ISS, NISS, and EISS classifications (Table 5) revealed that there were 757, 1,036, and 416 patients with ISS ≥ 25, NISS ≥ 25, and EISS ≥ 27. Both patients with ISS ≥ 25 and NISS ≥ 25 had significantly lower mortality rates compared to patients with EISS ≥ 27 (28.7% vs. 44.0%, OR 0.5 [95% CI 0.40–0.66], *p* <0.001 and 22.7% vs. 44.0%, OR 0.4 [95% CI 0.29–0.48], *p* <0.001, respectively). Additionally, the ICU admission rates for patients with ISS ≥ 25 and NISS ≥ 25 were 87.7% and 81.5%, respectively, and they were both significantly lower than that of patients with EISS ≥ 27 (92.3%). The patients admitted to the ICU with EISS ≥ 27 had significantly longer ICU LOS compared to patients who had ISS/NISS ≥ 25. Among these patients, those fatal patients had similar ICU LOS no matter which scoring system was applied. In contrast, the patients admitted to the ICU that survived with EISS ≥ 27 still had significantly longer ICU LOS compared to patients that survived with ISS/NISS ≥ 25. In terms of medical expenses, patients with NISS ≥ 25 had significantly lower cost of operation, cost of pharmaceutical, and total medical expenses in comparison with patients with EISS ≥ 27.

**Table 5:** Comparison of the demographics and detailed medical expenses of severely injured adult trauma patients classified according to the ISS, NISS, and EISS.

Variables	ISS≥25 n = 757 (I)	NISS≥25 n = 1036 (II)	EISS≥27 n = 416 (III)	*OR(95%CI) p*		*OR(95%CI) p*	
				I vs III		II vs III	
Age	51.5±18.8	52.1±19.0	52.4±19.3	—	0.443	—	0.820
Gender, n (%)							
Male	522(69.0)	709(68.4)	286(68.8)	1.0(0.78–1.31)	0.942	1.0(0.77–1.26)	0.907
Female	235(31.0)	327(31.6)	130(31.3)	1.0(0.77–1.28)	0.942	1.0(0.79–1.30)	0.907
Mortality, n (%)	217(28.7)	235(22.7)	183(44.0)	0.5(0.40–0.66)	<0.001	0.4(0.29–0.48)	<0.001
Hospital LOS (days)	20.6±19.6	19.5±18.3	20.4±22.3	—	0.916	—	0.449
ICU patients, n (%)	664(87.7)	844(81.5)	384(92.3)	0.6(0.39–0.91)	0.015	0.4(0.25–0.54)	<0.001
ICU LOS (days)	12.0±14.8	11.1±13.9	14.5±17.8	—	0.018	—	0.001
Mortality (ICU)	203(30.6)	217(25.7)	175(45.6)	0.5(0.41–0.68)	<0.001	0.4(0.32–0.53)	<0.001
ICU LOS (days)	7.4±9.0	7.6±9.1	7.2±8.6	—	0.890	—	0.659
Survival (ICU)	461(69.4)	627(74.3)	209(54.4)	1.9(1.47–2.47)	<0.001	2.4(1.88–3.12)	<0.001
ICU LOS (days)	14.0±16.4	12.3±15.1	20.6±21.0	—	<0.001	—	<0.001
Cost of operation	1148±1530	1022±1420	1332±1789	—	0.065	—	0.002
Cost of examination	578±612	540±580	606±665	—	0.481	—	0.078
Cost of pharmaceutical	919±1608	803±1472	1113±1787	—	0.066	—	0.002
Medical expenses	9652±10694	8683±9875	10933±12420	—	0.077	—	0.001

CI = confidence interval; ICU = intensive care unit; ISS = injury severity score; LOS = length of stay; EISS = exponential injury severity score; NISS = new injury severity score OR = odds ratio

**Fig 3:**
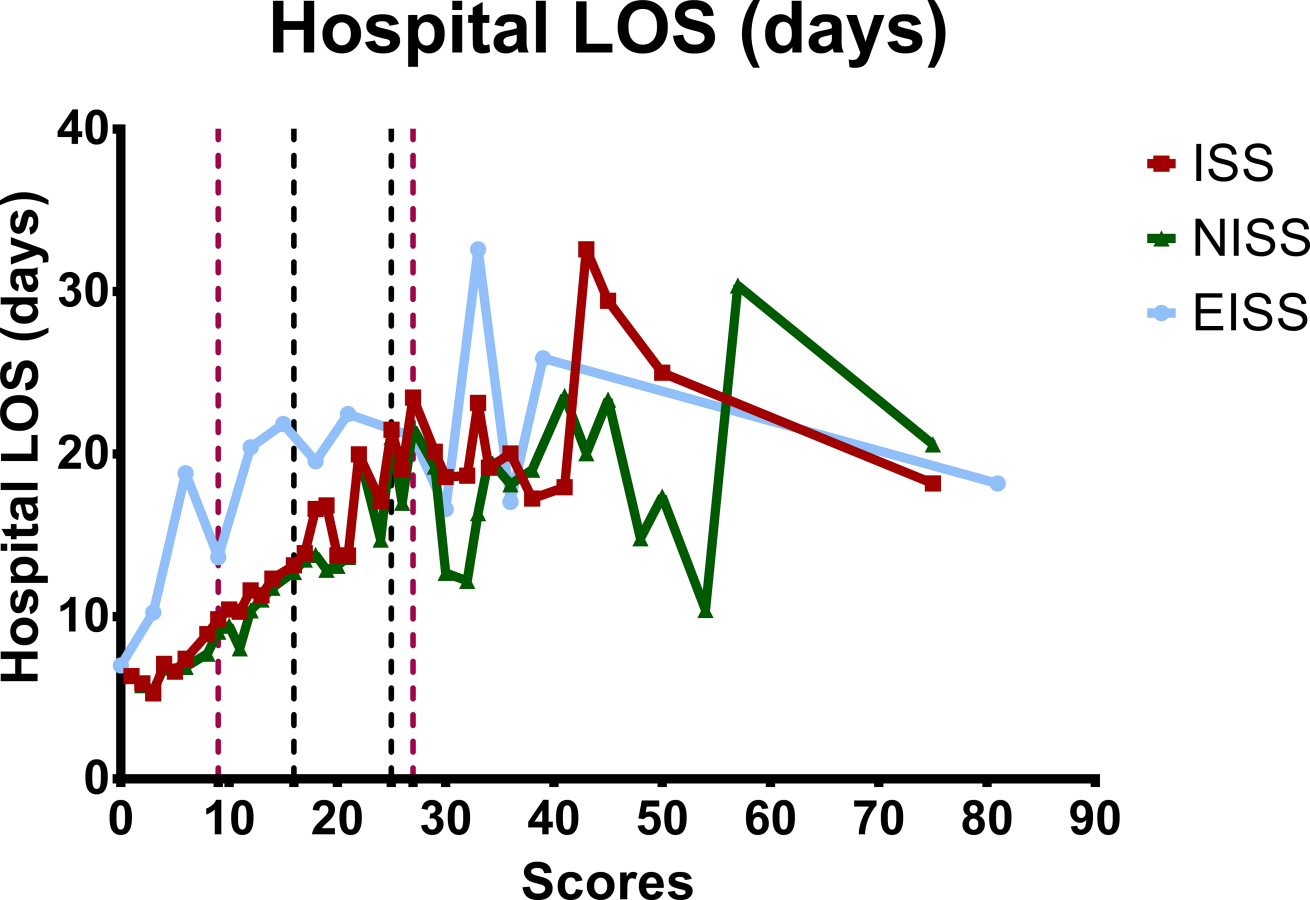
The hospital LOS-to-severity score relationships of all patients classified according to the ISS, NISS, and EISS.

**Fig 4:**
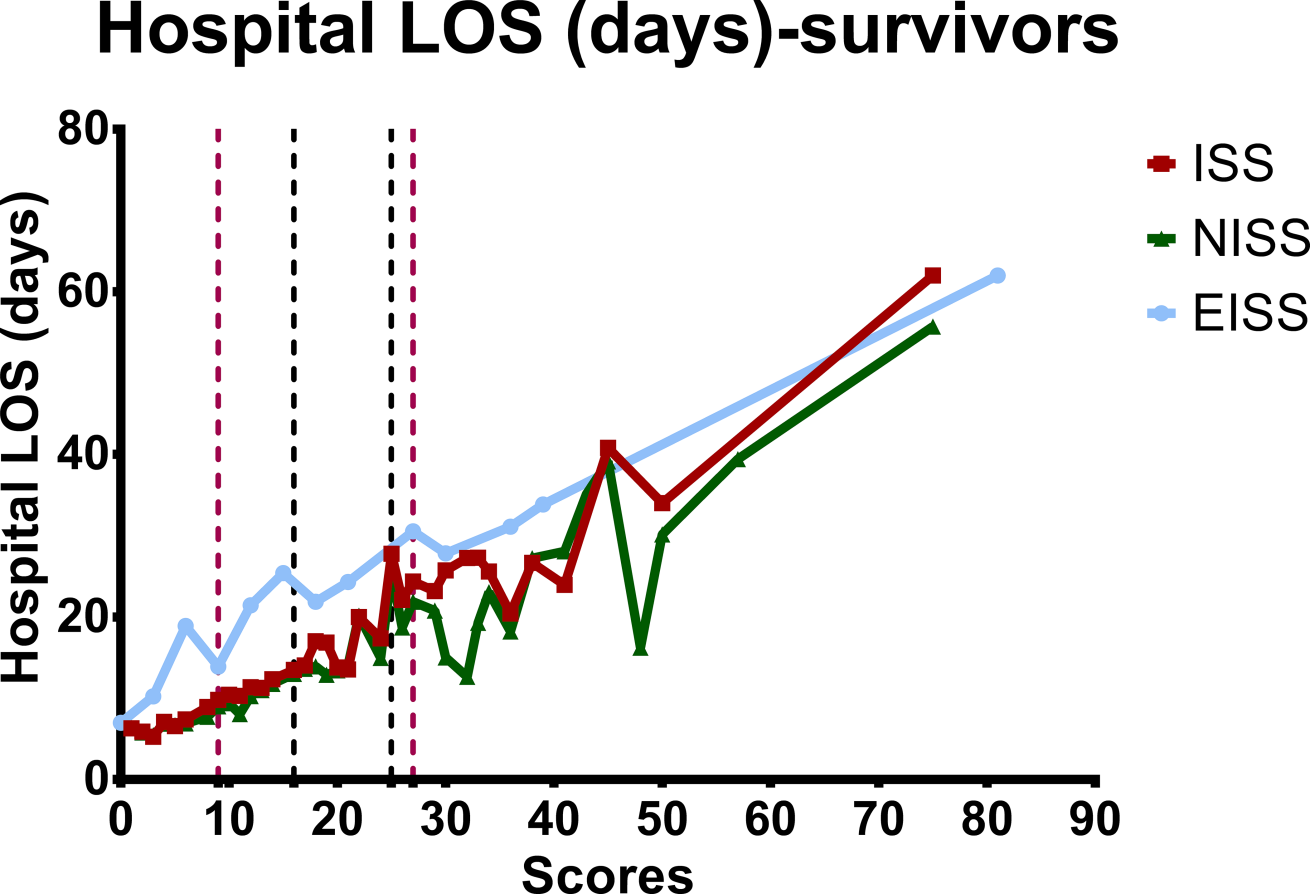
The hospital LOS-to-severity score relationships for survivors classified according to the ISS, NISS, and EISS.

**Fig 5:**
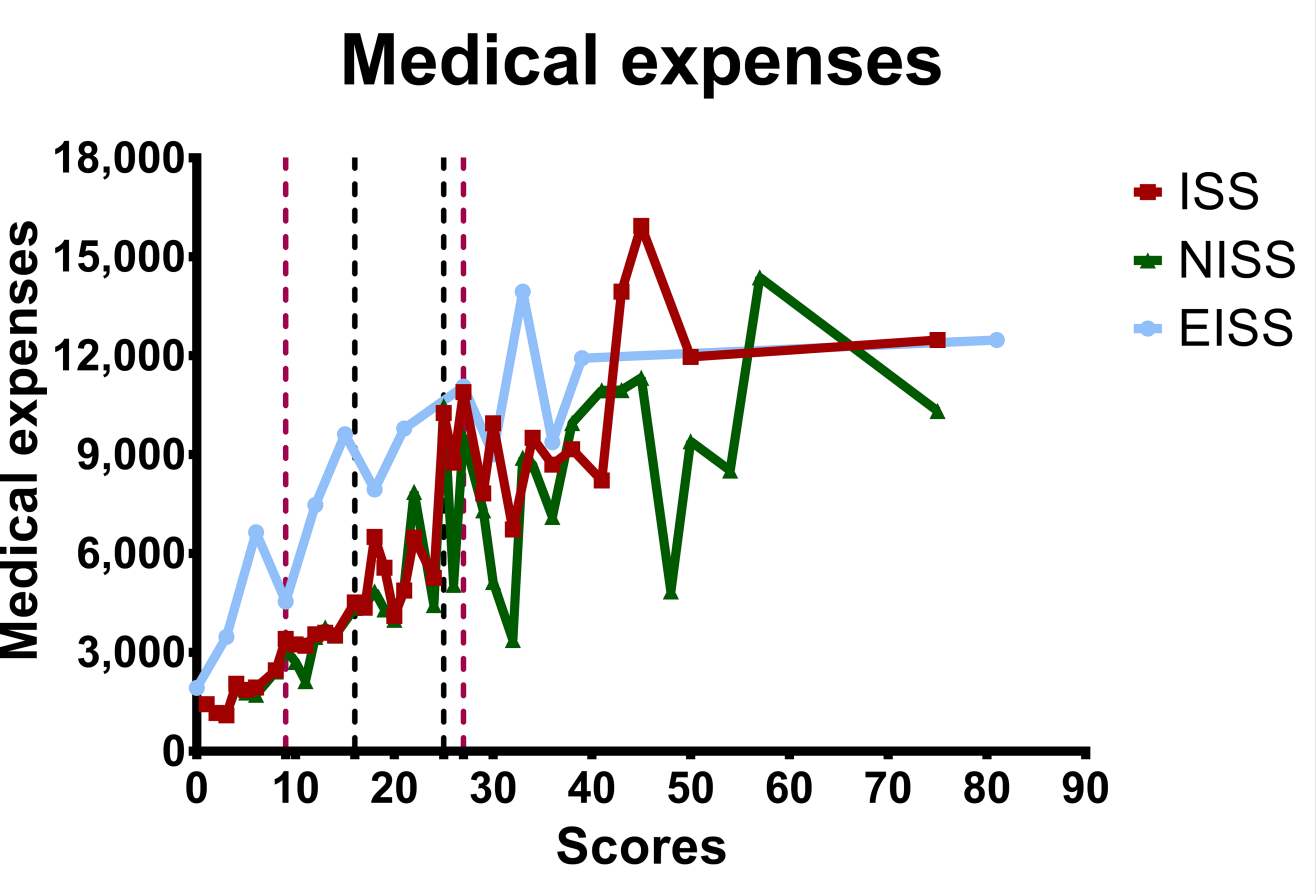
The total medical expenses-to-severity score relationships of all patients classified according to the ISS, NISS, and EISS.

The hospital LOS-to-severity score relationships of all patients and the survivors are summarized in Figs 3 and 4. The hospital LOS was well correlated with the severity score when ISS/NISS < 25 for patients classified based on ISS and NISS, especially if the patient sustained only mild injuries (ISS/NISS < 16). The EISS classification, on the other hand, did not correlate with or predict the hospital LOS well. The total medical expenses-to-severity score relationships of all adult trauma patients are summarized in Fig 5. The EISS did not correlate with and was not a predictor of the total medical expenses, whereas the ISS/NISS and the total medical expenses correlated well for patients with ISS/NISS < 16.

## Discussion

In this study, relevant demographics and detailed medical expense records of all adult trauma patients were collected and analyzed according to the ISS and the NISS classification system. In addition, we investigated the new EISS classification system. With the help of the survival rate-to-severity score relationship curve, two cut-off values were set (EISS: 9 and 27), and all patients were grouped into one of the following groups: patients with EISS ≥ 27, EISS of 9–26, and EISS < 9. The categorization correlated with that according to ISS and NISS, i.e., ISS/NISS ≥ 25, 25 > ISS/NISS ≥ 16, and ISS/NISS < 16. Not surprisingly, patients with EISS ≥ 27 had the highest mortality rate and medical expenses compared to those with EISS of 9–26 and EISS < 9, representing the patient group of greatest injury severity.

Moreover, we compared patients with ISS ≥ 25, NISS ≥ 25, and EISS ≥ 27, and discovered that patients with EISS ≥ 27 not only had the highest mortality, but also had the highest ICU admission rate and the longest ICU LOS among the three groups of patients. Additionally, only 416 patients had EISS ≥ 27, which were fewer than those with ISS ≥ 25 and NISS ≥ 25. In other words, EISS ≥ 27 included the patients that had the worst outcome, even when compared to those with ISS ≥ 25 or NISS ≥ 25.

Although the ISS seemed to be the most commonly used scheme to describe the severity of multiple trauma patients, it was still debatable whether the ISS or the NISS better differentiates mortality and poor outcome. A number of previous studies demonstrated that the NISS predicted mortality better than the ISS, and served as a better predictor of extended hospital LOS and ICU admission rate as compared to the ISS[4–8, 11]. Some studies observed better calibration, but equivalent discrimination with the ISS[12, 13]. Additionally, some studies found no advantages in using the NISS [14–18].

Wang et al. proposed the novel EISS in a retrospective cohort study, which reviewed data that comprised more than eight thousand patients from 2007 to 2012[3]. The examination and analysis in this study aimed to test the performance of the NISS and the EISS. These authors discovered that if the data sets were examined graphically, most of the survivors fell into the relatively lower EISS category when compared with the NISS, and that EISS better distinguished survivors from non-survivors. Furthermore, in the graph of the NISS against mortality, the authors noticed a very choppy and non-monotonic line, while the EISS mortality rates were distributed more closely to the auxiliary line in the graph of the EISS against mortality. Additionally, Wang et al. performed a formal statistical analysis to confirm the superior predictive power of the EISS over the NISS in terms of mortality. Although the ISS has already become a worldwide instrument for communication, while it would never be the case for the novel EISS (or even the NISS), they still concluded that the EISS provides better statistical characteristics, and provides a more accurate prediction of the prognosis and mortality when compared to the NISS. They therefore suggested replacing the ISS and the NISS with the novel EISS.

Our study established the cut-off values for the EISS further, and discovered that patients with high EISS had worse outcomes compared to those with either high ISS or NISS. However, in our study the EISS does not correlate well with hospital LOS or medical expenses. In addition, although the cutoff of 27 of EISS was selected for severe injury because of an obvious decrease in survival according to the survival rate-to-severity score relationship curve, there were fewer patients with EISS ≥ 27 than patients with ISS/NISS ≥ 25, therefore the mortality rate will increase relatively. This study also has several limitations. One major limitation is the retrospective design, which comes with an inherent selection bias. Secondly, the indications for ICU admission/discharge are not documented in our trauma registry system, and we also lack data regarding the circumstances of the injury. Another source of potential bias might be the exclusion of patients declared dead either on hospital arrival or at the accident scene, and those who were discharged against the advice. Finally, the population included in this study is limited to a single urban trauma center in southern Taiwan, which may not be representative of other populations.

## Conclusion

Based on this study, we conclude that EISS 9 and 27 might serve as two cut-off points regarding injury severity, and patients with EISS ≥ 27 represent the patient group with the greatest injury severity. These patients have the highest mortality rate, the highest ICU admission rate, and the longest ICU LOS compared to those with ISS ≥ 25 and NISS ≥ 25, suggesting that EISS ≥ 27 consists of the patient group with the worst outcomes.
